# Regulating RIPK1: another way in which ULK1 contributes to survival

**DOI:** 10.1080/15548627.2020.1783110

**Published:** 2020-06-24

**Authors:** Wenxian Wu, Björn Stork

**Affiliations:** Institute of Molecular Medicine I, Medical Faculty, Heinrich Heine University, Düsseldorf, Germany

**Keywords:** Autophagy, necroptosis, necrosome, RIPK1, TNF, ULK1

## Abstract

The mammalian ULK1 is the central initiating kinase of bulk and selective macroautophagy/autophagy processes. In the past, both autophagy-relevant and non-autophagy-relevant substrates of this Ser/Thr kinase have been reported. Here, we describe our recent finding that ULK1 also regulates TNF signaling pathways. We find that inhibition of autophagy or specifically ULK1 increases TNF-induced cell death. This autophagy-independent pro-survival function of ULK1 is mediated via the phosphorylation of RIPK1 at Ser357. RIPK1 is the central mediator of pro-inflammatory or pro-death signaling pathways induced by TNF, and ULK1-dependent phosphorylation regulates RIPK1 activation and distribution to different intracellular signaling complexes. Our results indicate that ULK1 exerts a cyto-protective function not only by initiating autophagy, but also by controlling RIPK1-mediated cell death.

Cells are continuously exposed to different intrinsic or extrinsic stresses. The ultimate fate of a cell is determined by the crosstalk of different cellular stress responses, such as autophagy, cellular senescence, apoptosis, or programmed necrosis. The execution of these pathways can result either in survival or in death. In recent years, there has been a substantial increase in interest concerning the crosstalk among these stress responses.

Autophagy represents an intracellular recycling pathway enabling the re-use of building blocks derived from long-lived or damaged proteins and organelles. It occurs at basal levels in most cell types and ensures cellular homeostasis. However, autophagic processes can also be actively induced under stress conditions such as nutrient depletion, protein aggregation, or infection with intracellular pathogens. Furthermore, autophagy can occur both as a nonselective recycling (bulk autophagy) and as a selective process, during which – for example – damaged organelles or intracellular pathogens are degraded. Both forms of autophagy are executed by autophagy-related (ATG) and non-ATG proteins, and both forms require the autophagy-initiating ULK1 complex. This complex is comprised of the Ser/Thr kinase ULK1 (unc-51 like kinase 1), ATG13, RB1CC1 (RB1-inducible coiled-coil 1), and ATG101. In the past decade, several substrates of ULK1 have been reported (Figure 1). Generally, these substrates can be classified into four subgroups: 1) components of the ULK1 complex, 2) components of the class III phosphatidylinositol 3-kinase (PtdIns3K) lipid kinase complex, 3) other autophagy-related proteins, or 4) non-autophagy-related proteins. Furthermore, several ULK1-interacting proteins have been characterized. Collectively, these ULK1 substrates and ULK1-interacting proteins already indicate that ULK1 – next to its essential role for the induction of autophagy – participates in several additional physiological processes, e.g. axon guidance during brain development, type I interferon production, ER-Golgi trafficking, regulation of chaperone function, mitosis, stress granule dynamics, WNT-CTNNB1 signaling, mineralocorticoid receptor signaling, cell migration and invasion, and non-autophagic regulation of cell death.

**Figure 1.**
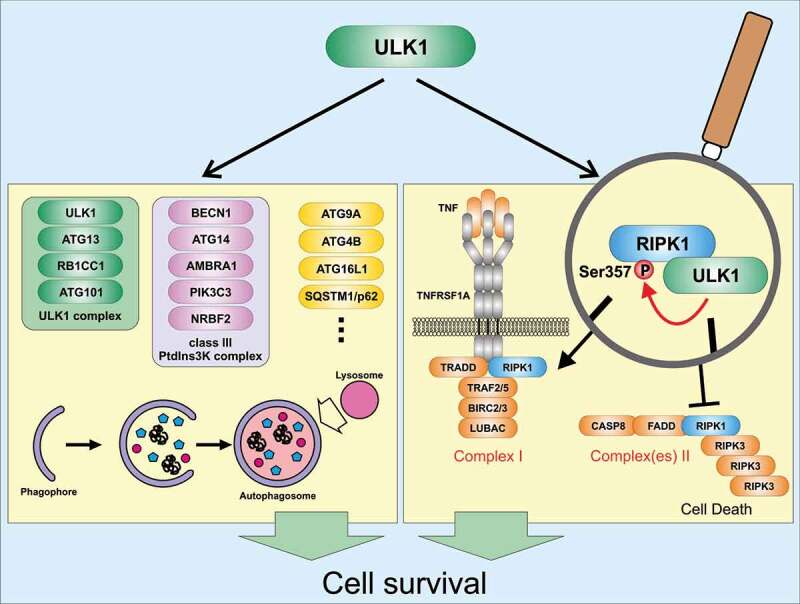
Cyto-protective signaling pathways of ULK1. During autophagy (left panel), several proteins become phosphorylated by ULK1. These substrates include components of the ULK1 complex itself, components of the class III PtdIns3 K complex, or several other autophagy-relevant proteins (please note that several additional autophagy-relevant substrates of ULK1 have been identified). Autophagy is initiated and fulfills a cyto-protective function. During TNF-TNFRSF1A signaling (right panel), ULK1-dependent phosphorylation of RIPK1 at Ser357 leads to reduced complex II assembly and stabilization of RIPK1 within complex I. Thus, cell death is prevented. AMBRA1, autophagy/beclin 1 regulator 1; ATG, autophagy-related; BECN1, beclin 1, autophagy related; BIRC2/cIAP1, baculoviral IAP repeat-containing 2; BIRC3/cIAP2, baculoviral IAP repeat-containing 3; CASP8, caspase 8; FADD, Fas (TNFRSF6)-associated via death domain; LUBAC, linear ubiquitin chain assembly complex; NRBF2, nuclear receptor binding factor 2; PIK3C3, phosphatidylinositol 3-kinase catalytic subunit type 3; RB1CC1, RB1-inducible coiled-coil 1; RIPK1, receptor (TNFRSF)-interacting serine-threonine kinase 1; RIPK3, receptor-interacting serine-threonine kinase 3; SQSTM1/p62, sequestosome 1; TNF, tumor necrosis factor; TNFRSF1A, tumor necrosis factor receptor superfamily, member 1a; TRADD, TNFRSF1A-associated via death domain; TRAF2/5, TNF receptor-associated factor 2/5; ULK1, unc-51 like kinase 1.

Necroptosis represents another cellular stress response and is the prototype of programmed necrosis. This process can be induced by several stimuli, such as ligand binding to death receptors, to Toll-like receptors, or to sensors of viral nucleic acids. Necroptosis induction downstream of TNFRSF1A (tumor necrosis factor receptor superfamily, member 1a) is probably the best characterized form of necroptosis induction. Generally, stimulation of TNFRSF1A by TNF can result in the activation of MAPK (mitogen-activated protein kinase) and NFKB/NF-κB (nuclear factor kappa-light-chain-enhancer of activated B cells) signaling pathways and the transcription of prosurvival genes and proinflammatory cytokines, or the activation of cell death pathways such as extrinsic apoptosis or necroptosis. The central mediator of these differential signaling outcomes is RIPK1 (receptor [TNFRSF]-interacting serine-threonine kinase 1). Within the TNFRSF1A-associated complex I, ubiquitinated RIPK1 fulfills a scaffolding function, and cell death is prevented. In contrast, upon dissociation of RIPK1 from complex I, different cytosolic RIPK1-containing complexes II have been described. Although the nomenclature of these complexes II is quite confusing for an autophagy researcher, these complexes II have in common that they support apoptotic or necroptotic cell death. When CASP8 (caspase 8) is absent or inactivated in complex II, kinase-active RIPK1 recruits and activates RIPK3 (receptor-interacting serine-threonine kinase 3), leading to the formation of a β-amyloid-like structure termed a necrosome. Activation of RIPK3 and RIPK3-dependent phosphorylation of MLKL (mixed lineage kinase domain-like) then result in MLKL oligomerization, translocation to the plasma membrane, and necroptotic plasma membrane permeabilization.

We aimed at investigating the crosstalk between the autophagy-initiating kinase ULK1 and the inflammation- and cell death-regulating kinase RIPK1 [1]. We observed that pharmacological or siRNA-mediated ablation of ULK1 increases TNF-induced cell death. Along these lines, TNF-induced cell death is reduced in *ulk1 ulk2* double-knockout (DKO) murine embryonic fibroblasts (MEFs) reconstituted with wild-type ULK1, but not in MEFs expressing a kinase-inactive variant of ULK1. We next analyzed formation of complex II/necrosome under ULK1-inhibiting conditions. We found that inhibition or deficiency of ULK1 increases the association of autophosphorylated RIPK1 with complex II/necrosome, and also the pro-necrotic ubiquitination of RIPK1 appears to be increased in these cytosolic complexes. In turn, RIPK1 association with complex I is increased in ULK1-expressing cells compared to *ulk1 ulk2* DKO MEFs. Collectively, these data suggest that ULK1 regulates RIPK1 distribution and activation in a kinase-dependent manner.

Accordingly, we investigated whether RIPK1 itself represents a substrate of ULK1. We performed in vitro kinase assays and identified several ULK1-dependent phospho-acceptor sites within RIPK1. Using different truncated variants of RIPK1, we only observed phosphorylation of the intermediate domain. By mutational analysis, we found that Ser357 of RIPK1 represents a major ULK1-dependent phospho-acceptor site. Although we observed ULK1 association with complex I, Ser357 phosphorylation of RIPK1 is only evident for complex II/necrosome but not for complex I. To ultimately prove that RIPK1 phosphorylation at Ser357 mediates the inhibitory effect of ULK1 on TNF-induced cell death, we expressed a S357A variant of RIPK1 in *ripk1* KO MEFs. The RIPK1 S357A-expressing cells exhibit increased RIPK1 autophosphorylation, MLKL phosphorylation, and a stabilized assembly of complex II/necrosome compared to cells expressing wild-type RIPK1. Furthermore, knockdown of ULK1 only sensitizes wild-type RIPK1-expressing cells for TNF-induced cell death, but not cells expressing the S357A variant. In summary, our data suggest that ULK1 negatively regulates TNF-induced cell death via the phosphorylation of RIPK1 at Ser357 (Figure 1).

Collectively, we propose a phosphorylation-dependent crosstalk between the major executioner kinases of autophagy and TNF-TNFRSF1A signaling. Our observations constitute another puzzle piece of the crosstalk between cellular stress responses in general and between autophagy and necroptosis in particular. It has previously been reported that autophagy dampens necroptosis by the turnover of RIP homotypic interaction motif (RHIM) domain-containing proteins, and also our data supporting a cyto-protective function of ULK1 point toward this direction. However, the complexity of this crosstalk is underscored by reports describing the pro-death function of ULK1 (i.e., the enhancement of PARP1 activity by nuclear ULK1), the scaffolding role of the autophagy machinery in balancing necroptosis and apoptosis (i.e., via the SQSTM1/p62 [sequestosome 1]-dependent recruitment of RIPK1), or the anti-autophagic function of RIPK1 (i.e., via the control of TFEB [transcription factor EB]). Nevertheless, what remains without doubt is that the autophagy-independent signaling properties of ULK1 are far from being completely elucidated.
